# Tracking Highly Similar Rat Instances under Heavy Occlusions: An Unsupervised Deep Generative Pipeline

**DOI:** 10.3390/jimaging8040109

**Published:** 2022-04-13

**Authors:** Anna Gelencsér-Horváth, László Kopácsi, Viktor Varga, Dávid Keller, Árpád Dobolyi, Kristóf Karacs, András Lőrincz

**Affiliations:** 1 Faculty of Information Technology and Bionics, Pázmány Péter Catholic University, Práter utca 50/A, 1083 Budapest, Hungary; karacs@itk.ppke.hu; 2 Department of Artificial Intelligence, Faculty of Informatics, Eötvös Loránd University, Pázmány Péter Sétány 1/C, 1117 Budapest, Hungary; kopacsi@inf.elte.hu (L.K.); vv@inf.elte.hu (V.V.); lorincz@inf.elte.hu (A.L.); 3 Laboratory of Neuromorphology, Department of Anatomy, Histology and Embryology, Semmelweis University, 1094 Budapest, Hungary; keller.david@med.semmelweis-univ.hu; 4 ELKH-ELTE Laboratory of Molecular and Systems Neurobiology, Eötvös Loránd Research Network, Eötvös Loránd University, 1000 Brussels, Belgium; dobolyi.arpad@ttk.elte.hu; 5 Department of Physiology and Neurobiology, Eötvös Loránd University, Pázmány Péter Sétány 1/A, 1117 Budapest, Hungary

**Keywords:** multi-object tracking, segmentation, edge enhancement, deep generative networks, computer vision, synthetic data generation

## Abstract

Identity tracking and instance segmentation are crucial in several areas of biological research. Behavior analysis of individuals in groups of similar animals is a task that emerges frequently in agriculture or pharmaceutical studies, among others. Automated annotation of many hours of surveillance videos can facilitate a large number of biological studies/experiments, which otherwise would not be feasible. Solutions based on machine learning generally perform well in tracking and instance segmentation; however, in the case of identical, unmarked instances (e.g., white rats or mice), even state-of-the-art approaches can frequently fail. We propose a pipeline of deep generative models for identity tracking and instance segmentation of highly similar instances, which, in contrast to most region-based approaches, exploits edge information and consequently helps to resolve ambiguity in heavily occluded cases. Our method is trained by synthetic data generation techniques, not requiring prior human annotation. We show that our approach greatly outperforms other state-of-the-art unsupervised methods in identity tracking and instance segmentation of unmarked rats in real-world laboratory video recordings.

## 1. Introduction

There are several fields with the need for automatic analysis of visual data. The breakthrough of deep learning methods is the most promising for this purpose. However, there is still a gap between human and machine vision precision in several image processing tasks. The handling of hardly visible contours is one of the challenging issues. Contours may be hardly visible due to the similarities of adjacent regions. The similarity between background and foreground objects may be due to animal camouflage, a disguise in nature to help animals hide in the surroundings. In addition, multiple nearly identical instances may occlude each other, which is another frequent scenario in diverse applications. There are many applications (including tasks in medicine and agriculture) where the observation of individual instances is required. Thus, accurate tracking is essential.

In medical and pharmaceutical research, visual tracking of treated animals is used for behavior analysis to detect the effects of drugs or other treatments. Rats and mice are commonly used as animal models [1,2]. There is a wide range of available strains with differences in needs (feeding, etc.), structural or physiological parameters [3] and in behavior patterns, and each study should choose the most appropriate strain model. We focus on data where mice/rats are very similar without any markers but may have received different medical treatments, and individual patterns should be analyzed. In typical setups, the camera is fixed, and the foreground segmentation from the background is feasible, which simplifies the segmentation process. However, handling the changes in body configurations and the heavy occlusions between the objects poses a significant challenge.

Identity (ID) tracking for behavior analysis can be further specified, introducing task-specific constraints on requirements and providing valuable assumptions. In medical research, the environment setup aims to ensure that instances are unable to leave the scene or hide behind obstacles because proper ID re-assignment cannot be guaranteed on re-entering. While avoiding ID switches (track switches) is crucial, the fixed number of instances can be exploited. We aim to avoid human annotation of hundreds of images, because it is time-consuming. In addition, automatic annotation-based learning methods are easy and fast to train and can be applied to new animals or data if they are slightly different.

**Figure 1:**
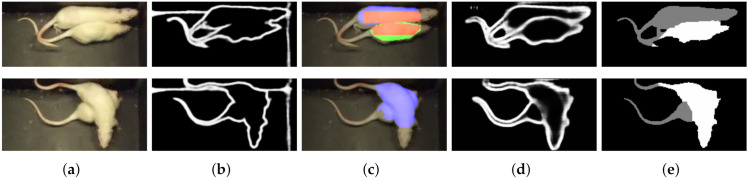
Images of the processing for two frames with occluding instances, with joint body segmentation using the Mask R-CNN [6] based method. From left to right: (**a**) input frame (**b**) original edge image (**c**) components from body part detection based on Mask R-CNN (**d**) our edge prediction giving rise to (**e**) our segmentation prediction exploiting information from body part tracking.

In the demonstration footage, the setup contains two adult female Wistar rats, which have identical white colors without any marker (Figure 1). The recording is from a single, downward-looking camera, and instance size changes are negligible. Our approach is to build a pipeline inspired by “Composite AI” [4,5], to provide reliable segmentation for identity tracking. Composite AI combines different learning architectures to achieve superior results and exploits human knowledge for improving the evaluations. In our case, for example, we use information about the number of components to be detected, among others.

In this paper, two main categories of contributions and procedures are presented.

1. We train edge detection deep networks based on synthetic data:
  - We propose a method for generating a synthetic dataset fully automatically (based on only frames with non-occluding instances), without any human annotation to train an edge detection network to detect the separating boundary between occluding identical objects, with a static background.
  - We extend the occlusion dataset to generate training data for edge enhancement by applying an unpaired CycleGAN, a well-known generative adversarial network (GAN) [7]. It is trained to learn the characteristics of the inner edges in overlapping instances in the output images of an edge detection step, where detected inner contours are discontinuous.
  - We train an inpainting network starting from the extended automatically annotated dataset. To improve the “continuity” of the boundary edges for precise segmentation, we generate inputs by occluding the separating edges of the highly similar instances.
2. We propose a combination of unsupervised deep networks to exploit edge information in addition to region-based methods.
  - For highly similar objects, we propose an instance segmentation method that combines the results of edge detection and the region-based approach presented in [8].
  - The propagation approach presented in [9] is used to track instances without switching in the test video.

The code will be publicly available at https://github.com/g-h-anna/sepaRats.

## 2. Related Works

Multiple methods address the problem of processing visual data of biomedical research of rodents, with computer vision. Deep Ethogram [10] identifies social interaction types, but cannot track identities; therefore, no individual patterns are available. DeepLabCut [11] utilizes keypoints and tracklet stitching methods in multi-object pose estimation and tracking. For a demonstration of identity prediction and tracking, they use a dataset of marmosets, with two animals in a cage, and a light blue dye is applied to the tuft of one of them, which is a distinctive visual marker. Moreover, for training, they used 7600 human-annotated frames, each containing two animals and 15 keypoints per animal. Currently, no pre-trained model is available on rodents.

Diverse approaches [12,13,14] are based on a module of DeepLabCut utilizing heavily prior human annotations of several frames, with multiple keypoints and keypoint connecting edges per animal. SLEAP uses the so-called human-in-the-loop method to achieve the necessary accuracy for pose estimation over the frames, which becomes continuous annotation in the prediction phase.

Idtracker, idtracker.ai, and ToxID [15,16,17] do not require prior data annotation; therefore, they are similar approaches as our proposed pipeline. However, for idtracker.ai the used C57BL/6J mice have a homogeneous black color, except their ears, which are visible in all poses, therefore representing a strong visual marker that may be utilized in feature learning of deep networks. On the other hand, idtracker.ai requires that the areas of the individuals barely change, a severe restriction for segmentation and separation of non-shape preserving instances. Rats are flexible and may take different forms in 3D, e.g., during an interaction or rearing [18] that introduce a large range of pixel-wise size variations.

**Figure 2:**
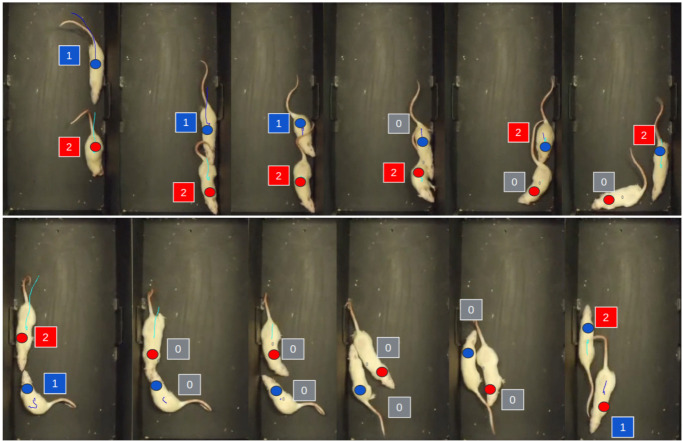
Two track switching examples using “ idtracker ” on our test data (cropped from the frames of the video). Ground truth labels are colored disks: blue (label 1), red (label 2). Predicted labels are within the squares. During occlusion, frame labels are lost (label 0), IDs and ground truth labels differ.

We applied idtracker, idtracker.ai, and ToxID on our data with various parameter settings. We found that several ID switches occur even for relatively simple videos. We show two such switches in Figure 2 for idtracker.

**Figure 3:**
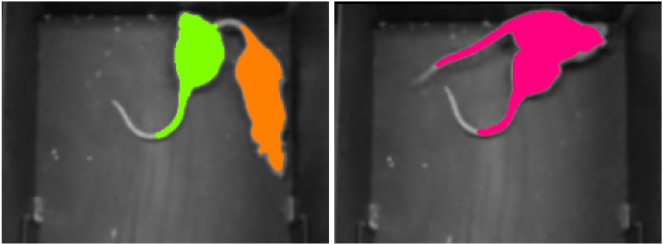
ToxTrac, which requires no preliminary annotation, was applied on our test sequences. Non-occlusion instances are usually well detected, but instances are not separated during occlusions: a single mask is provided.

ToxID method [17] segments individuals from the background and stitches traces across all frames. ToxID needs optimal illumination conditions, and along with idtracker.ai, it utilizes the fact that body shape and appearance do not change much. In addition, the similarity-based probabilistic texture analysis is not reliable for videos longer than about 20 min [19]. For ToxID an implementation is available, called ToxTrac [20]. We show a segmentation on a sample from our videos in Figure 3. ToxTrac does not aim to track the individuals without identity switches, but all tracks are saved for further statistical analysis, which would possibly be useful for joint behavior analysis.

**Figure 4:**
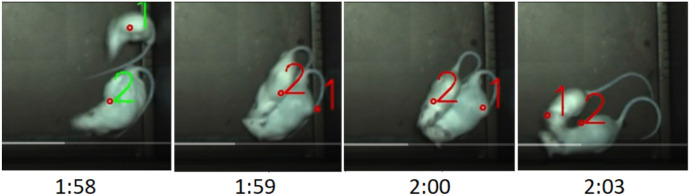
Some examples for frames selected from a demonstration video attached to [21] where ID tracking fails. The numbers 1 and 2 correspond to instance IDs. During episodes of significant overlaps multiple ID changes occur.

The method in Lv et al. [21] is built on YOLO and Kalman filter-based prediction for tracking bounding boxes of multiple identical objects (three white rats). In this method, human annotation is reduced by training on an automatically pre-calculated annotation of 500 frames, and requiring only some correction. However, the training dataset focuses mainly on non-occluding positions and the model is not prepared to track identities over episodes of significant overlaps. Object ID-s are switched often in the video (accessed on 16 October 2021, CC BY 4.0) provided by the authors, see a small illustration of a few frames in Figure 4.

In the past few years, there has been high interest and extensive research in deep networks [6,11,22,23,24,25,26,27] to improve instance segmentation over the combination of traditional image processing methods. When instances are highly similar and have few or no features and similar colors, regions are of little help, whereas edges still may be detected. In this paper, we utilize deep learning-based edge enhancement for instance segmentation.

When applied to complex scenes with multiple objects, basic edge detection methods such as Sobel [28] or Canny [29] use threshold values as parameters for balancing between being detailed enough, but not too noisy. When it comes to highly similar and occluding objects, these methods fail to detect crucial edges for instance separation.

There have been various attempts in deep learning instance segmentation under occlusion. Lazarow [30] proposed to build upon a Mask R-CNN [6] based architecture, but utilizes the differences of the objects. This approach achieves good results on benchmarks but fails to differentiate highly similar instances, such as the task of tracking rats.

In the case of highly similar and occluded objects, instance separation is often not possible using regional information only. When dealing with instances that have the same visual appearance (e.g., two white rats) edge information can help the segmentation of the overlapping instances. The deep DexiNed network [31] provides promising results for both inner and external edges. This architecture is based on the holistically-nested edge detection (HED) scheme [32], and with the fusion of the network layers, the produced output balances low and high-level edges. DexiNed was trained on the Barcelona Images for Perceptual Edge Detection (BIPED) database [31]. DexiNed predicts well-defined outer boundaries for instances, but the inner edges in overlapping positions are discontinuous. Deep generative networks can be exploited in such situations, that are capable of filling in the missing edge fractions. For filling in the missing parts of the edges inside the overlapping instances, we were inspired by the edge generation network called Edge-Connect [33]. Edge-Connect uses two GANs in tandem. It masks the images and trains an edge generator to estimate the edges in the masked regions. The second stage of the tandem learns the image completion task building upon the structure information of edges detected in the first stage. For our purpose the first stage of this double GAN is useful as our interest is in the separation of the animals. In the case of highly similar and occluded objects, accurate boundary information is hard to acquire.

Additional regional information such as the recognition of body parts may be invoked to find missing edges and to decrease the errors. Kopácsi et al. [8] proposed a method to detect body parts of rodents without the need for hand-annotated data. They used (i) various computer vision-based methods to find keypoints and body parts from foreground-background masks, (ii) synthetic data generation, and trained a Mask R-CNN [34] for keypoint detection and body part segmentation on unseen images. They applied the medial axis transformation and determined the head region, the body region, and the tail region using the geometrical properties of the rats.

## 3. Methods

The goal is high-quality instance tracking, i.e., the minimization of the number of erroneous switches. We overview our algorithm selection method. The individual computational steps are detailed in the main part of this section.

### 3.1. Overview of the Algorithm Selection Procedure

The optimization of segmentation is the first step of our heuristics. It is followed by a propagation algorithm.

We have tested multiple edge detection algorithms: the Sobel [28], the Canny [29] methods, and the trained BIPED model of the DexiNed [31] network. We dropped Sobel due to its low performance. We evaluated Canny and the BIPED model using F-score, precision and recall metrics [35]. Canny and the BIPED model produced similar results (see later). In turn, we decided on using the trainable method, i.e., DexiNed, as it had a greater potential.

We trained the Edge Detection in two ways: from scratch and with transfer learning starting from the BIPED model [31]. We used different ground truth edge thicknesses and the epoch number of the training procedure.

Selection was based on the performance on those frames where instances overlapped and the foreground mask was not connected. The model that had the lowest number of frames with a single connected foreground region was chosen. We refer to this model for the scratch and the transfer learning cases as SepaRats and BIPED-TL, respectively.

We used the selected models as follows. We estimated the foreground mask and generated edge images. If it was a single connected region then algorithm of Edge Completion was invoked. If the foreground mask had more than one regions then we combined it with the results of the *body parts* method [8] by means of higher level knowledge, the number of bodies (see later). In case of a single connected foreground region only the *body parts* method was used. The outcome is the *per-frame segmentation*. Per-frame segmentations were connected by a propagation algorithm [9].

**Figure 5:**
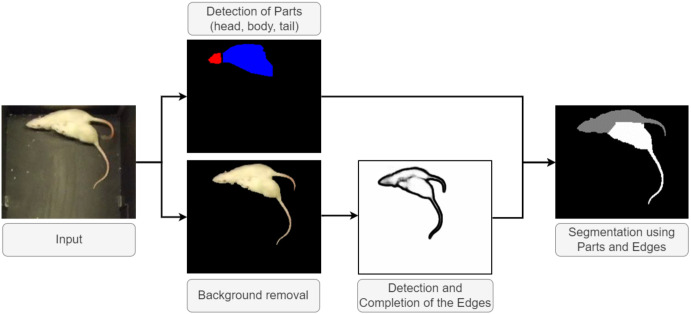
Sketch of the test pipeline for a single frame. Our aim is to maximize the segmentation precision within the frame to provide a strong basis for tracking. Body parts and edges are predicted and edge completion methods are invoked. A pre-processing provides the foreground masks and removes the background from the frame. A post-processing module combines the information and predicts the segmentation of the instances separately to enable identity tracking. Note the error in the last subfigure: the head of one of the animals is mislabeled. However, tracking remains error-free.

Below, we elaborate on the sub-components of our heuristics that give rise to the final evaluation pipeline. The overview of the main steps is depicted in Figure 5.

### 3.2. Pre-Processing

There are two animals in the scene. They are neither allowed to leave the cage nor able to hide, but while they are interacting, they can heavily occlude each other, making tracking challenging.

Thus, we differentiate between frames with disjoint and occluding object instances based on the foreground segmentation. For the latter case, i.e., frames with a single connected foreground region, for a given instance segmentation method we will differentiate between *frames with* separated and with *unseparated occluding object instances*.

We apply the DexiNed model (*BIPED model*), which was pre-trained on the first version of the BIPED dataset, as this performed better than later models (downloaded from https://github.com/xavysp/DexiNed, accessed on 21 March 2021). In what follows, we use interchangeably the following words: contour, edge, and boundary. These—in contrast to the geometric definitions—represent here the set of pixels marked as edges by DexiNed or other edge detection methods. We distinguish two types of boundaries: external boundary separates the background from the rest, i.e., from the occluding or non-occluding two animals. Inner boundary refers to the boundary within the foreground mask. This inner boundary may separate the animals, as seen later. During pre-processing, we use (a) the original frames, (b) the edge images predicted by BIPED model, and (c) the foreground masks as inputs.

As the background is static in our case, we can estimate the background by taking the mode of 2000 frames. Then, we can get the foreground via background subtraction. We also incorporate intensity histograms in the foreground estimation process to make it robust against slight illumination changes and reflections, the reflections on the cage parts, and the shiny urine the animals add to the scene occasionally. The drawback of this method is that parts of the tails might be lost. However, we can neglect the tails, as the rodent’s tail movement is not considered as a factor for tracking the rats. For frames with separated object instances the disjoint foreground masks are labeled with index values, which we define as ID values of the instances. The edges predicted by the BIPED model are outside the foreground mask. We define the edge-masks for each frame as the foreground mask and the instance contours detected by the BIPED model. The output of pre-processing are the foreground masks, the masks of the instances, the masked RGB instances, and the edge masks.

### 3.3. Augmentation

The automatic generation of annotated images is inspired by the idea in [36]. We used frames with occlusion-free samples for constructing overlapping instances. For the training of edge detection network, we also set the generating parameters to allow some non-overlapping positions, for the sake of comprehensive learning and to avoid overfitting on the significantly overlapping samples.

For each input frame, we generated five rotated samples evenly distributed along with adding a random correction.

We added two translated copies to each of them. The amplitude of translation was set according to a heuristic using the resolution of the frame, the sizes of the rats relative to the frame aspect ratio.

We applied these transformations to the RGB instances (region defined by the foreground mask) and for the masked edges, to create augmented RGB (aRGB) images and augmented ground truth (aGT) respectively. Unfortunately, if we put two correctly segmented single rats huddling or overlapping with each other for aRGB images, the shadows are different from the true occluding cases. Note that in 3D, the unoccluded instance is *above* the other one. This unoccluded instance will be called *the upper instance*. In particular, the shadow of the upper rat on the lower one is missing and it is the very relevant part, i.e., at the separating boundary. The foreground mask size for rats in RGB frames has a high impact. If it is small and excludes the shadows, then the shape is strongly damaged and becomes unrealistic. If it is large for an instance, then the shadow at the silhouette introduces an artifact that will be picked up by the deep network giving rise to failures on the real frames. The compromise is to minimize the damage to the shape while eliminating the artifacts by removing the unrealistic shadow from the RGB image with *inpaint-based blur*, as follows:

We define the separating boundary as the 1-pixel wide contour section of the upper rat within the mask of the overlapping instances, excluding the joint external boundary pixels. We use the inpaint function with the Telea algorithm [37] of the OpenCV package. Inpainting is invoked for the separating boundary, with a window radius of 7 pixels, to create a blur.

There is another pitfall that one needs to overcome: towards the external boundary, the blur is biased by the background pixels. We mitigated this by using an auxiliary background, we change the background to a rat-like one. We crop a region from the rats in a hand-picked frame of side-to-side contact. The cropped rat texture is resized to exceed the joint size of the overlapping instances and forms the background for us. This single resized image used for the augmentation of all RGB frames minimizes unrealistic artifacts for our case. Note that this particular approach is exploited only during inpainting the augmented RGB frames. Otherwise, the background is set to zero.

In aGT edge images only the edge pixels of the rats are non-zero. This is to minimize the influence of background prediction on the loss. To reduce the large number of background pixels, the area of the augmentations is set to a small, but sufficiently large square to enclose the overlapping instances. In our experiments, we used a square of 256 × 256 pixels.

The generated synthetic dataset of augmented inputs and aGT edge images are used in the training of all three networks presented in Section 3.4 and Section 3.5.

### 3.4. Training Edge Detection

**Figure 6:**
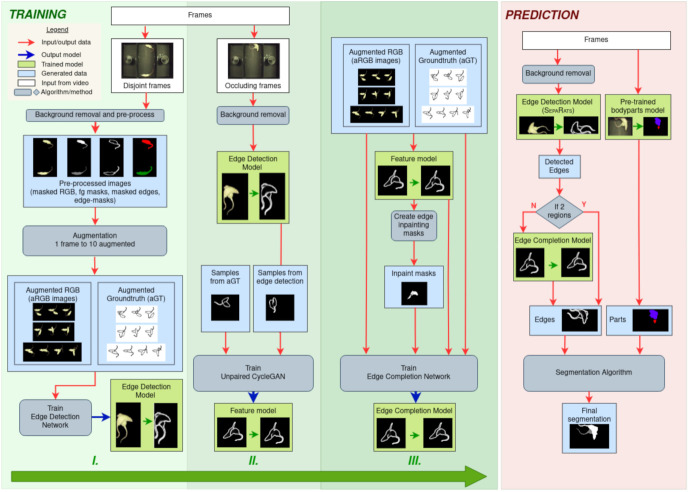
Illustration of the training and prediction pipelines. Three main blocks of the training pipeline: I. synthetic data generation and training Edge Detection model; II. training the Feature Model; III. extending synthetic dataset and training the Edge Completion model. Training is built upon the synthetic data generation. Overlapping inputs and augmented ground truth data are constructed from pre-processed frames with non-occluding instances. Trained Edge Detection network is applied on frames with occluding instances. The unpaired CycleGAN [7] generates training data on the synthetic dataset for training the Edge Completion network. The prediction pipeline applies the Edge Detection model on the foreground of the frames. If the detected edges are not separating the instances, the foreground mask is a single connected region and the trained Edge Completion network “extends” the edges inside the foreground mask. The segmentation algorithm predicts the final segmentation for each frame. The edge regions and the body regions detected by a pre-trained model [8] are combined to provide a reliable segmentation for identity tracking of the highly similar and markerless instances including during heavy overlaps. The proposed pipeline is completely automatic, no human annotation is required. For more details, see text.

We trained the network architecture presented in [31] with our synthetic dataset, described in Section 3.3. We present the illustration of this step in the Training I column of Figure 6. We used 73,328 input images with corresponding augmented ground truth edges. This trained model is the first deep network in the prediction pipeline. We trained multiple models for edge detection. We evaluated the models as described later, in Section 4.1 and picked the best in terms of separating the instances. This best model is called SepaRats.

### 3.5. Training the Edge Completion

In frames with occluding instances the foreground mask, apart form the tails, is a *single connected region*. The inner boundary as defined before separates the two instances. In what follows we refer to this part of the edge structure as the separating edge. If the separating edge is continuous, the upper instance has a closed contour corresponding to its mask boundary. We call the frames with overlapping instances *frames with* unseparated occluding object instances if the inner edge is discontinuous. Hence, in this case, there are less than two disjoint regions within the foreground mask.

We train an inpainting network to connect the edge endings in imperfect edge images (i.e., with unseparated object instances). For this step, we extend the augmented data (aGT and aRGB images) with corresponding imperfect edge images by transforming the continuous separating edges in the aGT edge images to non-continuous sections.

It is hard to formalize the characteristics of edge endings of missing contour parts for Edge Detection prediction with basic image processing methods and we use the unpaired CycleGAN proposed in [7] instead. To train a CyleGAN model, we apply our trained SepaRats Edge Detection model to frames with occluding instances of the training set. We randomly select 2200 predictions where the instances are unseparated, and an additional 2200 aGT edge images. We generate edge images with discontinuous separating edges using the trained CycleGAN model (denoted as Feature Model in Figure 6) for each aGT edge image, which provides input and desired output pairs for training an edge inpainting network, the Edge Completion Model illustrated in Figure 6, column Training II.

To train the edge completion network we use the same synthetic dataset as in the training of the Edge Detection network but extended with the imperfect edge images. We build our method on the generative adversarial network (GAN) proposed in [33]. This network uses three inputs: an RGB image, an inpaint mask that defines the area of missing edges, and the edge map to be completed. As opposed to [33], we do not apply masks to the RGB input of the generator network, because the aRGB image is always fully observable. We generate the inpaint mask in a way to ensure that no information is lost: it must cover the complete region of missing edges and at the same time exclude known boundaries: both the external contours, and the inner boundaries predicted by the CycleGAN Feature Model. We determine the mask of boundary pixels B by applying a threshold to the boundary image. Thus, the formula for calculating the inpaint mask M is as follows:

M=F∖B

where F denotes the foreground mask, and the operator ∖ stands for set difference. We note that in the prediction the inpaint mask can be generated in an analogous way, with the difference that the boundary image is provided by the Edge Detection model.

Because the primary goal of the edge completion model is to make the separating edge continuous for overlapping instances, this region needs to have a higher weight in the loss function than the non-boundary regions of the inpainting mask. Thus, we define the loss L of the discriminator for each frame as

$$L=BCELoss\left(M\circ E_{pred},\;M\circ E_{aGT}\right)+BCELoss\left(S\circ E_{pred},\;S\circ E_{aGT}\right)$$

where *BCELoss* stands for adversarial loss with Binary Cross Entropy [38,39] objective, and the operator ∘ denotes the Hadamard product [40], $E_{pred}$ and $E_{aGT}$ are the predicted edge image of the inpainting generator and the corresponding aGT edge image, respectively, and M denotes the inpaint mask. S is the mask of the separating edge. This mask of the separating edge consists of the missing part of the edge that we want to complete and is dilated to include a few pixel environment next to the edge. The Hadamard product restricts $E_{pred}$ and $E_{aGT}$ to the mask region while keeping the grey values [33].

For the discriminator, we modified the input with a re-scaling on the output of the generator as follows. The separating edges belong to the upper rat’s mask by construction, so we apply a heuristic: those edges that belong to the upper rat were given a weight of 1, whereas those that belong to the lower (occluded) rat, i.e., boundaries not related to the upper rat and thus containing only external boundary pixels of the overlapping instances are given a weight of 0.5. This weighting emphasizes the upper rat’s boundary in the discrimination procedure being critical for the closedness of this contour. We illustrate the training of the Edge Completion Model in column III of Figure 6.

### 3.6. Segmentation

See pseudocode for segmentation in Algorithm 1. The segmentation is the last step of the first stage of processing single frames. It is the post-processing of the information from body parts and edges. We focus on the occlusion of bodies and remove tails with a binary opening from the foreground mask of the input frame. The tails are not useful for the segmentation of the bodies and even represent a noise due to the various possible shapes.

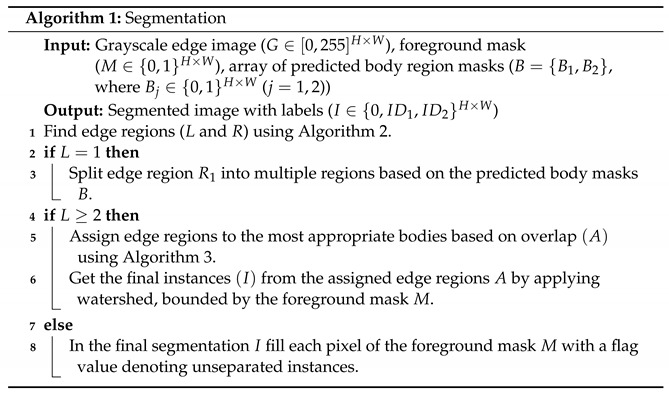


The combination of the information from edge and body parts detection consists of two main subroutines, as follows. First, we determine the regions corresponding to the detected edges. We use thresholding and binary morphological operations to transform predicted grayscale edges to a binary edge image. We used a randomly chosen subset of the training samples to select the threshold value. The output of the edge detection network is an intensity map, with higher intensities denoting higher certainty of the presence of an edge. The algorithm has two types of pitfalls (limiting factors). For high threshold values weak edges, such as the edge between the instances, get eliminated. In this case the foreground is made of a single connected region. For low threshold values two or more regions may appear. In addition, edge regions can cover considerable areas including a large part of the foreground, giving rise to high noise for the analysis following this thresholding. Upon visual inspection and considering the two limiting factors we set the threshold value θ to approximately 25% of the maximum (θ = 60 in the [0, 255] range) and set pixel values above (below) this threshold to 1 (0). This value is somewhat arbitrary and could be optimized if needed. We apply further transformation for thinning, and to reduce the noise introduced by this limit value, as follows. We call the resulting non-zero regions within the binary edge image *edge stripes*. The edge stripes are assumed to cover the contours and they can be up to 10 pixels wide depending on the output of Edge Complete and the parameters of the algorithm. To thin the stripes, we apply medial axis skeletonization of the scikit-image package [41] and calculate a midline (thresholded skeleton) of the edge stripes. The midline approximates the contour of the instance shapes. We add a dilation step for eliminating potential pixel errors and have a single connected region that splits the foreground mask into regions. Each region is labeled uniquely.

We provide the pseudocode in Algorithm 2.

**Figure 7:**
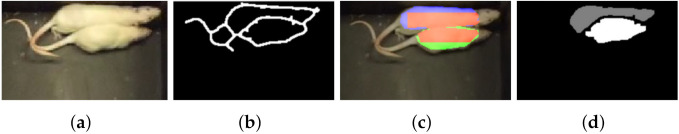
Initial labeling. (**a**) Grayscale input image (**b**) Dilated midline of the detected edges (**c**) Body parts prediction with initial labels shown by different colors (**d**) initial labels from (**b**). (**c**,**d**) are used for further segmentation steps in Algorithm 3.

**Figure 8:**
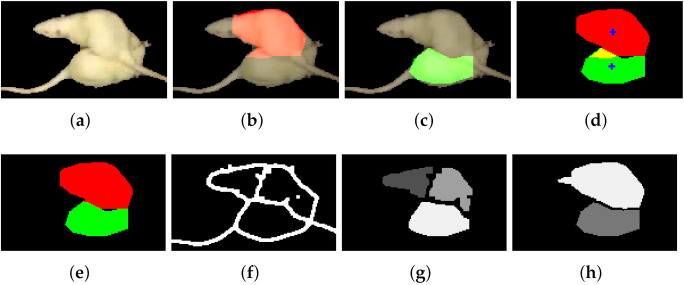
Due to identical appearances without any markers, region-based segmentation is often erroneous. If two bodies are predicted (shown in red and green), they are often overlapping, with non-realistic outlier contour points. We apply a centroid based method as a correction. (**a**) input; (**b**) first predicted body region; (**c**) second predicted body region; (**d**) both predicted body regions, yellow marks overlap, blue marks centroids; (**e**) after correction (each point in the intersection is assigned to the closest center of gravity); (**f**) edge midline Algorithm 2; (**g**) initial region labeling based on (**f**) and dilation; (**h**) region labeling combines (**e**,**g**) using Algorithm 3.

Second, we combine this information with the predicted body regions from parts detection, using the method proposed in [8]. In Figure 7 we illustrated the components of the creation of initial labeling for a frame. This approach is a Mask R-CNN-based [6] method, trained on synthetic data, without any human annotation. From the detected labeled regions (head, body, and tail), we use the predicted body regions in our combined method, while ignoring any heads and tails. Bodies may overlap, or have irregularities in the shapes due to outlier contour points. This would bias our method; therefore, a correction is needed, as follows. We filter this type of noise by formalizing the knowledge of the shape of the instances. We calculate the geometric center (centroid) for both bodies by taking the mean for the row and column coordinates. Pixels from the intersection area of the bodies are assigned to the closest center of gravity. See Figure 8 for our illustration.
**Algorithm 2:** Find edge regions **Input:** Grayscale edges ($G\in {\left[0,255\right]}^{H\times W}$) **Output:** Edge-based labeling for regions ($R_{i}\in {\left\{0,1\right\}}^{H\times W}$) (i=1,2,⋯,L),      number of edge regions (*L*)**1** Threshold the grayscale edges *G* with θ = 60**2** Apply morphological closing to get binary edges (B).**3** Apply medial axis transformation to the binary edges *B* to get its midline (M).**4** Find distinct regions (R) (*L* pieces labeled with 1…,L) bounded by the midline *M*.

For cases when rats heavily occlude each other, the separation of the bodies is hard, often a single joint body region is predicted only. In Figure 7 the main components are illustrated for calculating the labeled image for a frame. We assign predicted body regions and edge regions to each other, based on the ratio of overlap. If a single predicted body region is assigned to all edge regions, then body information is ignored, and initial labeling is created using only in the edge regions.

If two predicted body regions are detected, each edge region is unioned with the belonging body mask, and unique labels are set for united regions. Details can be found in Algorithm 3. We have no information about instance identities within a single frame; therefore, for simplicity, we label the bigger region with label 1, the smaller one with label 2. For any pixel which would have both labels, we set the label of the background, $L_{bg}=0$. These labels form the initial markers for a watershed algorithm [41,42] to fill the foreground mask and return the instance segmentation.

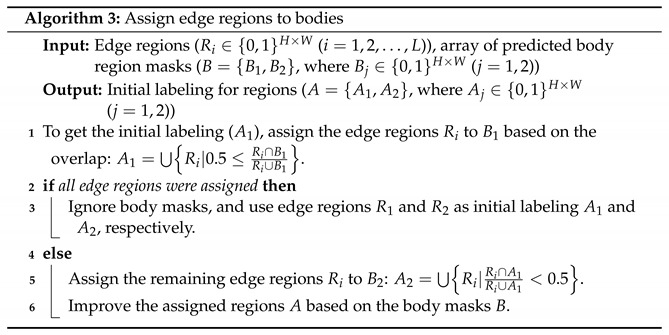


If the segmentation map contains only a single instance ID then the pixels of the foreground region are marked by a flag indicating that Algorithm 2 was not able to separate the instances. This flag may be used for further processing either using information propagation methods, such as in [9] or to call a human annotator to examine and correct.

### 3.7. Frame Sequence Propagation

Our main concern is to avoid ID switches during tracking. To improve the prediction for a single frame, we use the information from neighboring frames. We used the propagation approach presented in RATS [9], with slight modifications. Due to the different characteristics in appearance between the predictions of the Mask R-CNN model used by RATS and our proposed instance segmentation method, the parameters had to be tuned. We also incorporated optical flow (OF) [43] in the propagation to improve its reliability. We propagated the frames which were preserved by the rule-based method of [9], by shifting the pixels with the corresponding OF. However, this works well for short sequences, but the successive application of pixel-wise OF transformation can smear instances masks for longer timeframes. For this reason, we only used the propagated masks to guide the bipartite matching process [44] and used the original predictions for the final output.

## 4. Results and Discussion

We used 15,400 frames for training from a sample video. These frames correspond to about an 11-min video, which is very short when compared to the length of the necessary recordings needed for pharmaceutical studies.

We used a hand-annotated representative subset of 3600 frames from the surveillance video recorded during biological research aiming at measuring animal behavior under medical treatments. This is the ground truth data for the evaluation. These images were not used for training. The ratio of images with heavily occluding instances is around 15%, and the distribution of these subsequences in the complete sequence is nearly uniform.

For medical research, it is crucial to reliably maintain the identity of the instances. Therefore, to evaluate the performance of the tracking, we use the track switches (TS) metric from [9], which measures the number of instance ID switches during tracking. When only trajectories were available, such as in the case of ToxTrac [17] and idtracker [15], the track switch evaluation was conducted by human observers.

To evaluate segmentation quality we use Intersection over Union *IoU*, also known as the Jaccard index [45]. This metric gives us information about the amount of mislabeled pixels. For a prediction *P* and a ground truth *G*, *IoU* is calculated as $IoU=\frac{P⋂G}{P⋃G}$. For benchmark measures (F Mean, F Recall), we used the same ones as in [9], with the implementation taken from the Davis Challenge [46].

We compare our results to previously published literature methods, which are designed for tracking without human annotation: idtracker [15], idtracker.ai [16], and the ToxTrac [17] implementation of ToxID.

**Figure 9:**
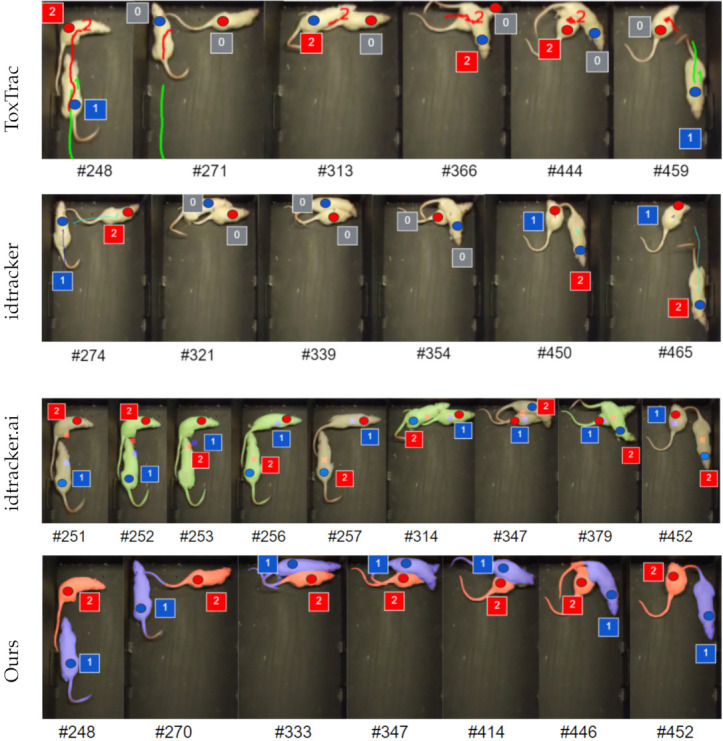
Illustrative visualization of methods compared. We use the track switches (TS) metric from [9], which measures the number of ID switches during tracking. Errors are shown for ToxTrac, idtracker, idtracker.ai in the first three rows. The results of our method (with SepaRats) for the most challenging frames are given in the last row showing no errors. The small circles denote the ground truth IDs (blue for instance with ID 1 and red for instance with ID 2), the squares denote the predicted ID labels. Gray denotes “lost ID”, missing ID predictions.

As ToxTrac and idtracker do not provide per-frame segmentation masks, we relied on the GUIs and output videos by the authors and the output videos we generated. In the case of idtracker.ai, we used the codes published on the web (https://gitlab.com/polavieja_lab/idtrackerai_notebooks/-/tree/master/ (accessed on 28 October 2021)) to export the segmentation masks and compared the pixels numerically to our automatically predicted segmentation. For all methods, we determined the number of identity switches using the videos. Results are presented in Table 1. An illustrative comparison is shown Figure 9.

The mean *IoU* values are similar for the last three methods of Table 1 and are considerably higher than that of the idtracker.ai method, the third row in the table. The methods may predict a single connected foreground region (column *number of frames with lost IDs*). While the BIPED & Parts approach produces only two such frames, still the number of track switches is higher. This is due to discontinuities in the separating edge: BIPED can produce uneven (smaller and larger) regions and that noise can spoil the propagation algorithm. This demonstrates that track switches, the primary failure of ID tracking, may occur even with a relatively high *IoU*, 0.833 in this case.

For the sake of completeness, in the following subsections, we present the evaluations of the edge detection modules and the combined segmentation algorithm of our proposed pipeline.

### 4.1. Edge Detection and Completion

**Figure 10:**
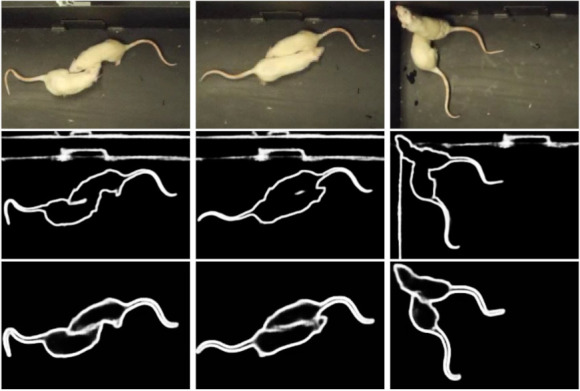
Edge detection results. (**Top row**): three different RGB frames. (**Middle row**): corresponding edge detection with BIPED DexiNed. (**Bottom row**): our Edge Detection results, with SepaRats model. Our Edge Detection predicts well-defined grayscale edges in the inner sections of the boundaries that can be thresholded.

We trained the Edge Detection network with the augmented dataset with transfer learning on the original BIPED model, named BIPED-TL, and also from scratch, named SepaRats. We trained these two models for 15 epochs on the 73,328 images using about 10% of the images for validation. The aGT method (described in Section 3.2) performed the best from a set of different augmentations that we created. For example, we used one pixel wide ($aGT_{1px}$) and two pixel wide ($aGT_{2px}$) contours of the foreground masks, but their performances were lower. For evaluating Edge Detection and Edge Completion we used the frames with occluding instances and neglected the easy non-occluding cases. In Figure 10, we show illustrative edge detection results for three frames with occluding instances.

The two most promising models (SepaRats and BIPED-TL) are in a close range, which means that the model exploited the training data to learn the main characteristics of the input, which is significantly different from the original, BIPED dataset.

**Figure 11:**
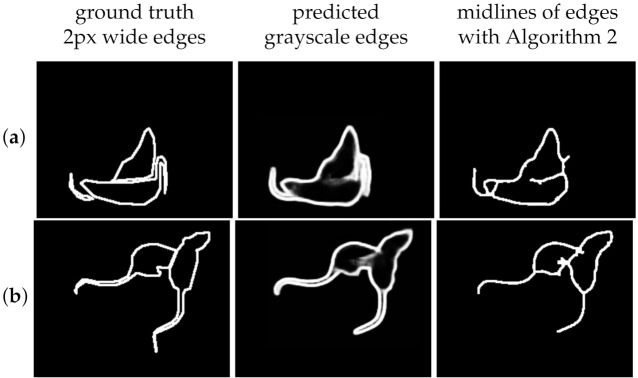
Illustration for the edge measurements. The F-score of the predicted grayscale edges in (**a**) (F = 0.6069) is better than the F-score of (**b**) (F = 0.5610). The predicted edges for (**b**) are distorted, but the extracted midline allows segmentation for both instances separately, as described in Algorithm 1.

The task of identity tracking is highly tolerant to distortions and shifts of the estimated edges as opposed to the gaps in the edges. Traditional measures treat the precision of the pixels of the edges instead of their continuity. Continuity is most important in the inner part of the boundary for separation and tracking. Figure 11 illustrates the significance of continuity. Therefore, to compare edge detection methods in terms of the addressed task, we apply both direct and indirect evaluation approaches, similarly to [47].

To select the most promising approaches, we consider the traditional measures (recall, precision, and F-score). F-score is calculated as $\frac{2PR}{P+R}$, where *P* stands for precision, *R* for recall between the ground truth and predicted contours. We show the results of the edge-related methods for F-score, precision, and recall [35] values using the implementation of the DAVIS challenge [45].

Edge error measures are instructive in selecting promising approaches. Both BIPED-TL and SepaRats improved the edge prediction of inner edges, compared to the pre-trained BIPED model and the Canny algorithm. The results are presented in Table 2. F-score and recall increased to 0.4362 and 0.4781, respectively, using Edge Completion after SepaRats.

Although an edge prediction that exactly matches the ground truth is a sufficient condition for instance segmentation, it is not definitely necessary. As opposed to a non-distorted separating edge that does not close the contour of the instance (with higher *F*, *P*, and *R*), a closed but distorted contour of the “upper rat” (with lower *F*, *P*, and *R* values) is sufficient for segmentation and reliable identity tracking. Therefore, as an indirect evaluation of the edge detection methods we rely on the results in Table 1 (the segmentation and tracking quality of the combined algorithm using the parts based regions and the regions from the edge detection models). The most important evaluation method is the number of track switches. The results are in line with the ones in Table 2.

In addition, we evaluated the segmentation quality for all three models. There is only a slight difference between the results of SepaRats and BIPED-TL models when used for segmentation and tracking. This indicates that the training had sufficient data to achieve optimal weights not only by transfer learning from the BIPED model but also from scratch.

We calculated the mean values for the 471 frames with occluding instances. When evaluating per frame segmentation quality, the IDs of the segments are not yet known: the ID assignment happens in a later stage of the processing pipeline. The segmentation step only considers information from a given frame. Thus, for the case of two instances, we can compare them to the ground truth in different ways and compute the corresponding *IoU* values. We take the maximum of these values.

If only tails occlude, the method presented in Algorithm 1 provides a reliable segmentation since the bodies have disjoint contours. Therefore we evaluate *IoU* values for the complete instance masks and also with leaving the tails using the body part detection method, if available. We present the calculated recall, precision, and F-score in Table 3.

The BIPED model requires the complete input frame, not only the foreground. In the prediction, the background elements and noises are significantly present. To compare the methods we apply the dilated foreground mask on the BIPED edge images.

Results in Table 3 show that for critical frames with overlapping instances trained models perform better.

### 4.2. Ablation Analysis

Key components of the segmentation compute (a) the edge based regions and (b) the parts based regions. We evaluated the combined segmentation method of Algorithm 1 with both components (edges and parts) in the prediction. For comparisons we also evaluated the two components separately using all three edge models, i.e., SepaRats and BIPED-TL, and BIPED as a baseline. Table 4 shows the Jaccard index (*IoU*) [45], the average values of recall, precision, and F-score for the predicted segmentations.

We evaluate per-frame segmentation in these ablation studies without using any information from the neighbouring frames and for those frames when the method could separate the instances. The number of frames with unseparated object instances was between 84 and 106 for the ablated methods with the models trained on synthetic rodent datasets (cca. 17–22% of occluding frames), but the frames were different for each method.

Since dropped frames are critical, the aim of this evaluation is to show the precision of the segmentation for the frames with successfully separated object instances. Results are shown in Table 4. We have seen in Section 4.1 that although SepaRats is slightly weaker in terms of edge precision than BIPED-TL, it performs better in the segmentation quality of frames with successfully separated object instances.

## 5. Conclusions

Instance tracking algorithms are relevant in diverse fields as they have a broad range of applications from the improvement of detection to prediction. We address the problem of avoiding trajectory switches in tracking identical, markerless instances. This is a frequently emerging need in biological research when doing automatic behavior analysis of two individuals, e.g., when they have different medical treatments.

Our algorithm consists of a carefully designed algorithmic pipeline that selects isolated instances followed by an augmentation step serving several deep learning methods. Deep learning tools of the pipeline detect the whole body, parts of the body, the motions of the bodies using optical flow, and in particular, the edges of the bodies: the detected and enhanced boundaries are combined with predicted body region information of the method presented in [8]. We also applied the propagation algorithm presented in [9] on the segmented frames to improve the reliability of tracking.

Algorithms with the intent of completely avoiding human annotation exist in the biological domain. We used our algorithm on close-to-white colored rats without any specific keypoints. We compared our algorithm to three state-of-the-art identity tracking methods of similar kinds. We showed that no trajectory switches occurred for our method, whereas the competitive methods made several mistakes. We showed that the prediction masks of our pipeline outperform single frame segmentation of idtracker.ai—the only method that provides such information out of the three that we investigated—in the critical occluding situations.

The robustness of our method could be increased by augmentation on illumination and changes in the foreground. These extensions should also increase the robustness of the method for other datasets.

## Figures and Tables

**Table 1:** Comparison of segmentation-based trajectory tracking of methods which do not require prior data annotation, on our test data of 3600 frames. TS denotes the track switches metric from [9], which measures the number of ID switches during tracking. Lost ID: there are less than two different foreground labels in the frame. For ToxTrac and idtracker, per frame segmentation masks are not available. Superscript 1 marks that the result is computed by means of the GUIs of the cited method, the best we could do for comparisons. Gray highlight: Mean of the *IoU* values for all frames. For benchmark measures, we used the same ones as in RATS [9].

ID Tracking Results							
Approach	Num. of TS.	Num. of Frames with Lost IDs	IoU Mean	IoU & F Mean	IoU Recall	F Mean	F Recall
ToxTrac [17]	9	267 ^1^	N/A	N/A	N/A	N/A	N/A
idtracker [15]	8	1055 ^1^	N/A	N/A	N/A	N/A	N/A
idtracker.ai [16]	10	1485	0.5556	0.604	0.59	0.652	0.746
BIPED & Parts	4	2	0.833	0.871	0.978	0.908	0.985
SepaRats & Parts	0	0	0.846	0.883	0.994	0.921	1.000
BIPED-TL & Parts	0	0	0.845	0.883	0.994	0.921	0.999

**Table 2:** Evaluation of different edge detection methods on our test sequence of 3600 frames, on the inner part of the overlapping objects. F-score is a support value for our decision, for more details, see text. Bold fonts indicate the results for the proposed models.

Edge Detection					
Approach	Deep Learning	Epoch	Transfer Learning	F Mean	Recall Mean
Canny	N	-	-	0.3614	0.3838
BIPED	Y	24	No	0.3510	0.3033
**BIPED-TL **	**Y**	**8**	**Y**	**0.4526**	**0.4902**
**SepaRats**	**Y**	**4**	**N**	**0.4328**	**0.4676**

**Table 3:** Evaluation of the segmentation method, combining edge based regions (from the trained models SepaRats and BIPED-TL, and the baseline BIPED) and regions from body part detection. The 471 frames of the test dataset with occluding instances were used for evaluation. *IoU* is calculated without tails. Mean values over the instances are shown for each metric. For all 471 frames there are at least two connected foreground regions with different labels, i.e., both ID labels are present on each prediction.

Segmentation				
Edge Detection Model	IoU Mean	Recall Mean	Precision Mean	F Mean
SepaRats	0.8087	0.8870	0.9025	0.8888
BIPED-TL	0.7872	0.8779	0.8883	0.871
BIPED	0.7455	0.8320	0.8359	0.8247

**Table 4:** Comparison of per-frame segmentation on the 471 frames with occluding instances. In lines (1), (2), and (3) we present the results for the combined segmentation method with both modules: Edge Detection and body parts regions. In (4), (5), (6), and (7) we show the quality of the segmentation for the modules separately. These values are marked by * to indicate that evaluation is only performed for the frames where two different foreground labels were predicted. Right-most column: number of dropped frames for the different methods.

Segmentation					
Method	IoU Mean	Recall Mean	Precision Mean	F Mean	Num. of Frames with Unseparated Instances
(1) SepaRats& parts	0.7991	0.8895	0.8897	0.8830	0
(2) BIPED-TL & parts	0.7872	0.8779	0.8883	0.8710	0
(3) BIPED & parts	0.7456	0.832	0.8359	0.8247	0
(4) parts	0.8295 *	0.9147 *	0.9008 *	0.9047 *	85
(5) SepaRats	0.7603 *	0.8657 *	0.8689 *	0.8559 *	84
(6) BIPED-TL	0.7826 *	0.8725 *	0.8915 *	0.8694 *	106
(7) BIPED	0.7925 *	0.8653 *	0.8920 *	0.8722 *	384

## Data Availability

The data presented in this study will be openly available in a repository, in case of acceptance.
